# Age-related changes in attentional control across adolescence: how does this impact emotion regulation capacities?

**DOI:** 10.3389/fpsyg.2014.00111

**Published:** 2014-02-12

**Authors:** Kathrin Cohen Kadosh, Lauren C. Heathcote, Jennifer Y. F. Lau

**Affiliations:** ^1^Department of Experimental Psychology, University of OxfordOxford, UK; ^2^Psychology Department, Institute of Psychiatry, King's College LondonLondon, UK

**Keywords:** adolescence, anxiety, attention, cognitive development, face processing, emotional expressions, emotion regulation

## Abstract

This study set out to establish the novel use of the go/no-go Overlap task for investigating the role of attentional control capacities in the processing of emotional expressions across different age-groups within adolescence: at the onset of adolescence (11–12 year-olds) and toward the end of adolescence (17–18 year-olds). We also looked at how attentional control in the processing of fearful, happy, and neutral expressions relates to individual differences in trait anxiety in these adolescent groups. We were able to show that younger adolescents, but not older adolescents had more difficulties with attention control in the presence of all faces, but particularly in the presence of fearful faces. Moreover, we found that across all groups, adolescents with higher trait anxiety exhibited attentional avoidance of all faces, which facilitated relatively better performance on the primary task. These differences in reaction time emerged in the context of comparable accuracy level in the primary task across age-groups. Our results contribute to our understanding of how attentional control abilities to faces but in particular fearful expressions may mature across adolescence. This may affect learning about the environment and the acquisition of behavioral response patterns in the social world.

## Introduction

Adolescence is a period of life which brings on profound changes in the social environment, physical growth, and substantial hormonal changes (Blakemore, 2008; Burnett et al., 2011). Behaviorally, adolescence is also associated with an increasing emotional variability (relative to childhood and adulthood), which, in at-risk individuals, may precipitate even more extreme emotional responses (Somerville et al., 2011). It is therefore not surprising that adolescence is also a period in which affective symptoms of anxiety and depression often emerge. These emotional extremes are common, with approximately 1 in 4 adolescents exhibiting increased levels of worries and anxiety. Moreover, adolescent anxiety predicts lifelong persistent mental health problems (Pine et al., 1998; Beddington et al., 2008).

Several compelling hypotheses have pointed to typical neurocognitive changes in adolescence as underlying this increased emotionality and tendency to experience anxiety. The adolescent brain exhibits protracted brain maturation (Giedd et al., 1999; Harris et al., 2011; Lebel and Beaulieu, 2011; Petanjek et al., 2011), a developmental process which enables substantial cognitive development and allows for a more flexible response to changing social environments (Scherf et al., 2012). Part of the considerable program of cognitive development is the acquisition and application of sophisticated strategies for regulating responses to emotional and social stimuli such as faces (Blakemore, 2008; Scherf et al., 2012; Cohen Kadosh et al., 2013a; Goddings et al., in press). However, prolonged acquisition periods can also place some individuals at risk for developing maladaptive and persistent emotional responses in the face of new, co-occurring psychosocial challenges (Paus et al., 2008). It remains to be determined which individual differences act as potential risk factors during adolescence. An effective and proactive way of regulating emotions is the use of attentional control to flexibly direct attention away from or toward emotionally salient stimuli (Todd et al., 2012). As attentional control engages lateral prefrontal cortex activity, one of the last regions to fully mature—and not till early adulthood (Gogtay et al., 2004; Mills et al., 2014)—it is plausible that gradual maturation of attentional control in the presence of emotional stimuli across adolescence can explain changes in emotional responses in this period. Moreover, these developmental changes in attentional control could bring out individual differences in anxiety-proneness.

The first purpose of the current study was to establish the novel use of the Overlap task (Bindemann et al., 2005) for investigating the role of attentional control capacities in the processing of facial expressions during adolescence. We then used this task: (i) to investigate whether this aspect of attention control emerges across adolescence, by comparing whether the capacity to direct attention away from various emotional faces differs in early relative to late adolescence, and (ii) to investigate the extent to which attentional control in the presence of emotional faces correlate with anxiety symptoms in adolescence. Identifying the timeframe in which attentional control is fully functioning and mature, and the corresponding “sensitive periods” in which difficulties are still observed, is crucial to identify optimal periods to administer emotion regulation interventions (Cohen Kadosh et al., 2013b). Moreover, gaining a better understanding of how individual differences in adolescent anxiety relate to attentional control capacity allows one to selectively target these interventions to at-risk individuals.

While adolescent attention control has been studied in “cold” cognitive tasks (Crone, 2009), fewer studies have investigated the developmental trajectory of attention deployment in the presence of emotional (including threatening) stimuli. Given that social-affective contexts are more likely to unleash adolescent difficulties in emotion regulation, we hypothesized that there would be more dramatic changes associated with attention when this is taxed by the presence of emotional distractors. Most research on attention processing to emotional stimuli has relied on two main experimental paradigms: the *emotional Stroop task* (Stroop, 1935) and the *dot-probe paradigm* (MacLeod et al., 1986). The emotional Stroop task is a modified version of the color-naming Stroop paradigm, where the emotional valence as well as the color of the printed word is varied (e.g., participants may be required to name colors of emotionally negative words). A negative attentional bias is inferred in this paradigm if participants take longer to name the color of the negative word (knife) relative to the neutral word (table). However, this paradigm has been criticized, as the increased reading time might well result from late processes that are unrelated to the early, automatic capture of attention. For example, it has been suggested that the observed delay might reflect the effortful avoidance of processing potentially threatening stimuli in the first place (de Ruiter and Brosschot, 1994). In the dot-probe paradigm, two stimuli are briefly shown simultaneously in two separate locations (one threatening stimulus and one neutral stimulus), followed by a small dot-probe in the location just occupied by one of them. An attentional bias toward threat would then be inferred if participants were faster to respond to a dot that is presented in same location as the threatening stimulus. While the dot-probe paradigm has the advantage of requiring an overt response to a neutral stimulus, it is somewhat less clear whether the observed response bias is due to faster engagement with the threat stimulus, or slower disengagement from it, thereby blurring the underlying time course. In addition, presenting two stimuli simultaneously may introduce additional attentional noise, with participants switching back and forth between the two stimuli.

Given these methodological limitations of the emotional Stroop and the dot-probe task, in our study we used the Overlap task (Bindemann et al., 2005) to assess age-dependent differences in attention disengagement from emotional stimuli. The Overlap task is a simple classification task, where participants focus on a central go/no-go signal, before, on go trials, responding to a peripheral line target. The Overlap task was first developed by Bindemann and colleagues, who used photographs of neutral faces, meaningful objects, and printed names. The present study offers the first use of this task with emotional stimuli in a developmental sample. The Overlap task offers an improvement on the Stroop and dot-probe task in that participants focus on a central emotional stimulus (a face), but still make an overt response to a neutral target (peripheral lines).

Our emotional stimuli were human faces displaying a range of emotions. Human faces are highly salient stimuli, which provide a plethora of social information, including identity, emotional expression, and direction of eye gaze. Previous research has shown that the ability to process emotional expressions accurately develops slowly up until childhood and adolescence (Thomas et al., 2007; Cohen Kadosh, 2011a). Moreover, these developmental trajectories vary as a function of emotional valence, for example, happy expressions are categorized correctly from an early age (Durand et al., 2007), while the accuracy of fearful expression categorization shows a linear improvement throughout adolescence (Thomas et al., 2007).

In the current study, we used the Overlap task to investigate age-related changes and anxiety-associated differences in attention control to different facial expressions. Based on known changes in adolescent emotionality, we predicted age-related differences in the capacity to modulate attention to face emotion stimuli. We tentatively predicted that this would be stronger for threatening (i.e., fearful faces) compared to non-threatening (i.e., neutral, positive faces) stimuli.

Secondly, we sought to investigate whether there were differences in attention control to emotional stimuli as a function of trait anxiety. Anxious adults have been found to struggle with attention control (Bishop, 2009), which in the presence of threatening stimuli, can result in an automatic orienting bias toward threats (see Bar-Haim et al., 2007). However, data extending measurement of these biases in adolescents have been less clear-cut. Using the dot-probe task, some studies have reported increased vigilance toward threat cues, but others have found the opposite or no differences associated with anxiety. These discrepancies could be explained by exposure time of the emotional stimulus: under shorter exposure times, anxious participants tend to show greater vigilance toward emotional stimuli, but under longer exposure times, these same participants may show avoidance of these stimuli (Bar-Haim et al., 2007). Given the nature of the Overlap task, and the ongoing presence of the emotional stimulus during performance of the primary task, we tentatively predicted that adolescents reporting higher levels of trait anxiety would show an attentional avoidance of the emotional stimulus, manifesting as quicker reaction times to the primary task.

Of note, the Overlap task also includes both go and no-go trials. Inclusion of these trials allows us, as a secondary aim, to also look at response inhibition, that is, the mechanisms involved in inhibiting an overt motor response in the no-go trials. As with attentional disengagement, we were able to look at age- and anxiety-associated differences in response inhibition and whether these varied across different emotional expressions.

## Methods

### Participants

Thirty young and thirty older adolescents from two school year groups were recruited from local schools as part of a large recruitment drive for a multiple-experiment project. The younger group had an average age of 11.5 years, *SD* = 0.50 years; (22 females), while the older group had an average age of 17.0 years, *SD* = 0.35 years (10 females). All participants had normal or corrected-to-normal vision and reported no history of neurological or psychological illness (this was determined via self-selection out of study, and report from teachers and parents). Informed consent was obtained from the primary caregiver and informed assent was obtained from the adolescents prior to testing. Participants received an Amazon voucher (£10) for participating in the experiment. The study was approved by the local ethics committee (Department of Experimental Psychology, University of Oxford).

### Experimental stimuli

For the Overlap task (Bindemann et al., 2005), we created a stimulus set from 9 color photographs of female faces (3 women × 3 emotional expressions (fearful, happy, neutral) that were selected from the NimStim set^1^. All pictures were cropped to show the face in frontal view and to exclude the neck and haircut of the person. For the face + target stimuli, a fixation cross was superimposed onto the face between the two eyes, and two black peripheral lines were presented on each side of the face. In total, 36 different stimuli [3 women × 3 expressions × target right or left of the face × green/red fixation cross (go/no-go trials)] were created. When viewed at a distance of approximately 70 cm, the three faces subtended 9.8° × 10° of visual angle. The two lines were presented at 22° of visual angle, subtending 0.2° × 2° of visual angle. Note that we used only female faces in the current study in order to keep any task-irrelevant stimulus variation at a minimum. This approach was chosen, as it has been shown that facial identity serves a reference frame for interpreting emotional expressions (Ganel and Goshen-Gottstein, 2004; Cohen Kadosh, 2011a) and that sex changes influence identity processing (Ganel and Goshen-Gottstein, 2002).

### Experimental procedure

The Overlap task was presented on a 17” Dell laptop (1024 × 768 pixel resolution), with a standard keyboard layout. Participants were sat approximately 70 cm away from the computer monitor, and were instructed to focus on the centre fixation cross when it appeared on the screen.

**Figure 1:**
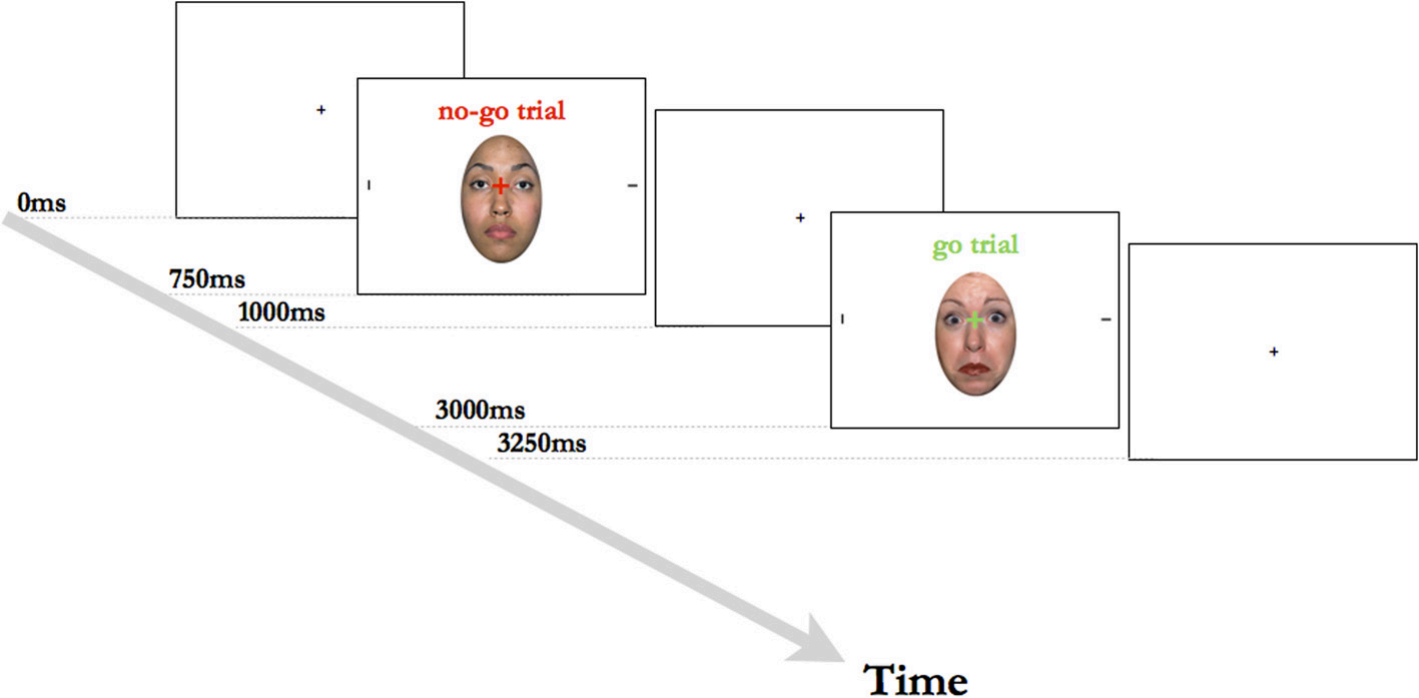
**Two example trials from the Overlap task**. The central fixation cross was green on go trials or red on no-go trials. The horizontal target line was equally likely to occur on the left or the right side of the face, with the vertical line always appearing on the opposite side.

Each trial began with a central black fixation cross on a white background, being presented for 750 ms. The fixation cross was then replaced for 250 ms by the face + target stimulus, with a red or green fixation cross super-imposed onto a face flanked by two peripheral black lines. The color of the fixation cross indicated whether the trial was a go trial (green color) or a no-go trial (red color). During the go trials, the participant's task was to indicate which of the two lines on either side of the face was presented horizontally. Participants were instructed to indicate the location of the target stimulus via a button press on a keyboard, with the key for the letter “L” corresponding to a target on the right side of the face and the key for the letter “A” corresponding to a target on the left side of the face. During no-go trials, participants were instructed not to respond and to wait for the next trial to begin. The face + target stimulus was followed by a white screen with black fixation cross, which was displayed for 3000 ms, or until a response was registered (see also Figure 1). Each session began with 12 practice trials (6 go trials, 6 no-go trials), with each emotional expression being shown 4 times. The practice was followed by 4 blocks of 36 trials with a ratio of 2:1 go (24) to no-go (12) trials, with each facial expression (fearful/neutral/happy) being shown an equal number of times in the trials. Additionally, we created three pseudo-randomized variations of the task to ensure that each emotional expression and trial type varied systematically throughout the blocks. Participants were encouraged to take self-paced breaks in-between testing blocks. Reaction times (RTs) to go trials formed our primary dependent measure and only RTs within a time range of 150–3000 ms post stimulus presentation were included in the analyses (this range covered at least 75% of all trials for each participant). We note that we chose a cut-off of 3000 ms post stimulus presentation, to avoid including responses that happened at the beginning of the subsequent trial. A second independent measure was accuracy to go trials. Finally, as a secondary aim, we also investigated accuracy rates during no-go trials (i.e., the correct inhibition of a motor response) to examine whether there were age group or anxiety-linked differences in response inhibition.

### Anxiety questionnaire

All participants completed the trait anxiety scale of the State-trait anxiety inventory for children (STAIC) (Spielberger et al., 1970). The STAIC features a self-report scale for measuring distinct anxiety concepts of trait anxiety. The trait anxiety scale consists of 20 trait anxiety statements that ask participants to indicate on a scale from 1 to 3 how they feel *in general* (1 being rarely anxious and 3 being often anxious).

## Results

### Trait anxiety levels

We compared the mean scores for each age group for the trait anxiety questionnaire scale, using *t*-tests. The two groups differed in their trait anxiety scores [*t*_(58)_ = −3.03, *p* = 0.004, *d* = 0.79], with the younger adolescents scoring on average 33 points (*SD*: 7.9) and the older adolescents scoring on average 39 points (*SD*: 8.2), with higher scores signifying higher trait anxiety levels. The individual scores for the trait anxiety scales were therefore included as a covariate in the analysis of the behavioral results.

### Overlap task

Mean reaction times (RTs) were calculated for correct go trials only. These were subjected to a 2-Way ANCOVA with the within-subject factor “expression” (fearful, happy, neutral), and the between-subject factor “age group” (younger adolescents, older adolescents), and individual trait anxiety scores as a covariate. The main effect of age group was significant [*F*_(1, 57)_ = 5.03, *p* = 0.029, η*p*^2^ = 0.081], as was the interaction between expression × age group [*F*_(2, 114)_ = 3.55, *p* = 0.032, η*p*^2^ = 0.059]. See Table 1 for all effects, and Table S1 for the same analysis without the covariate. Both sets of results yielded generally similar patterns of age differences and age-by-expression differences.

**Table 1:** **Analysis of covariance (ANCOVA) for the factors expression × age group with anxiety as a covariate**.

**Effect**	**Reaction times**	**Accuracy rates go trials**	**Accuracy rates no-go trials**
Expression	*F*_(2, 114)_ = 1.22, *p* = 0.300, η*p*^2^ = 0.021	*F*_(2, 114)_ = 0.49, *p* = 0.616, η*p*^2^ = 0.010	*F*_(2, 114)_ = 2.89, *p* = 0.060, η*p*^2^ = 0.048
Age group	***F***_(1, 57)_ = **5.03**, ***p*** = **0.029**, η***p***^2^ = **0.081**	*F*_(1, 57)_ = 0.12, *p* = 0.727, η*p*^2^ = 0.002	*F*_(1, 57)_ = 0.68, *p* = 0.412, η*p*^2^ = 0.011
Age group × anxiety	*F*_(1, 57)_ = 0.001, *p* = 0.974, η*p*^2^ < 0.001	*F*_(1, 57)_ = 0.007, *p* = 0.932, η*p*^2^ < 0.001	*F*_(1, 57)_ = 0.749, *p* = 0.390, η*p*^2^ < 0.001
Expression × age group	***F***_(2, 114)_ = **3.55**, ***p*** = **0.032**, η***p***^2^ = **0.059**	*F*_(2, 114)_ = 1.19, *p* = 0.307, η*p*^2^ = 0.020	***F***_(2, 114)_ = **3.34**, ***p*** = **0.039**, η***p***^2^ = **0.055**
Expression × anxiety	*F*_(2, 114)_ = 1.80, *p* = 0.170, η*p*^2^ = 0.031	*F*_(2, 114)_ = 0.41, *p* = 0.661, η*p*^2^ = 0.009	***F***_(2, 114)_ = **3.40**, ***p*** = **0.037**, η***p***^2^ = **0.056**
Expression × age group × anxiety	*F*_(2, 114)_ = 0.348, *p* = 0.707, η*p*^2^ = 0.006	*F*_(2, 114)_ = 2.59, *p* = 0.080, η*p*^2^ = 0.044	*F*_(2, 114)_ = 0.449, *p* = 0.639, η*p*^2^ < 0.001

Bold font indicates significant values.

**Figure 2:**
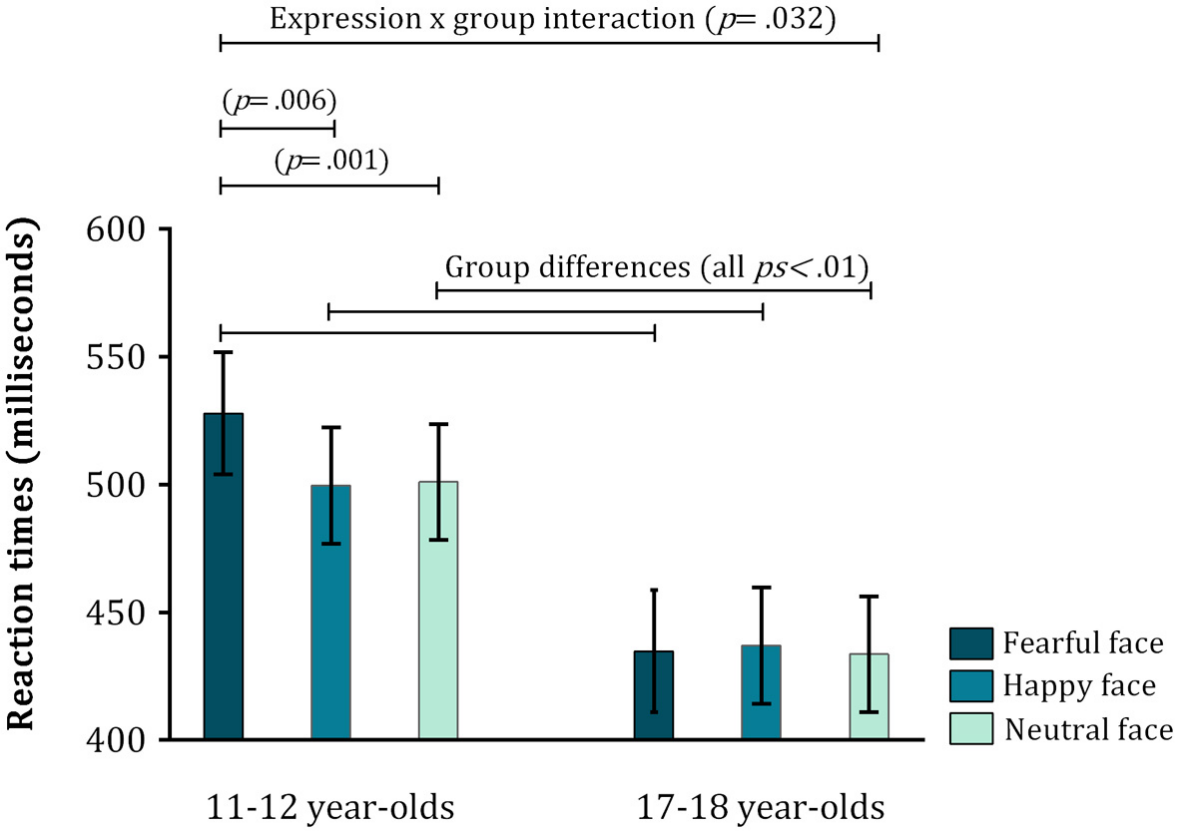
**Mean reaction times (in milliseconds) for the go trials in both age groups**. Error bars indicate 1 standard error of the mean. An expression × group interaction indicated a significant increase in reaction times toward targets that followed the presentation of fearful faces in the younger adolescents.

As seen in Figure 2, older adolescents were generally quicker to disengage attention from emotional faces than younger adolescents – and this was true for all three expressions [fearful face: *t*_(58)_ = 3.28, *p* = 0.002, *d* = 0.86; happy face: *t*_(58)_ = 2.69, *p* = 0.009, *d* = 0.70; neutral face: *t*_(58)_ = 2.69, *p* = 0.009, *d* = 0.70]. Instead, what determined the source of the interaction, was that the simple main effect of expression was significant in the younger group [*F*_(2, 114)_ = 7.2, *p* = 0.001, η*p*^2^ = 0.12], but not in the older group [*F*_(2, 114)_ = 0.083, *p* = 0.92, η*p*^2^ < 0.001]. Planned comparisons of the different emotional expressions in this age group showed that the younger participants took significantly longer to disengage from a fearful face toward the target in comparison to a happy face [*t*_(28)_ = 2.87, *p* = 0.006, *r*^2^ = 0.093] or a neutral face [*t*_(28)_ = 3.41, *p* = 0.001, *r*^2^ = 0.11]. There was no difference in disengagement speed for happy vs. neutral faces [*t*_(28)_ = 0.20, *p* = 0.848, *r*^2^ < 0.001] (Figure 2). Finally, trait anxiety correlated significantly with mean reaction times for all three emotional expressions across both age groups (fearful face: *r* = −0.268, *p* = 0.038; happy face: *r* = −0.320, *p* = 0.011; neutral face: *r* = −0.272, *p* = 0.035).

**Figure 3:**
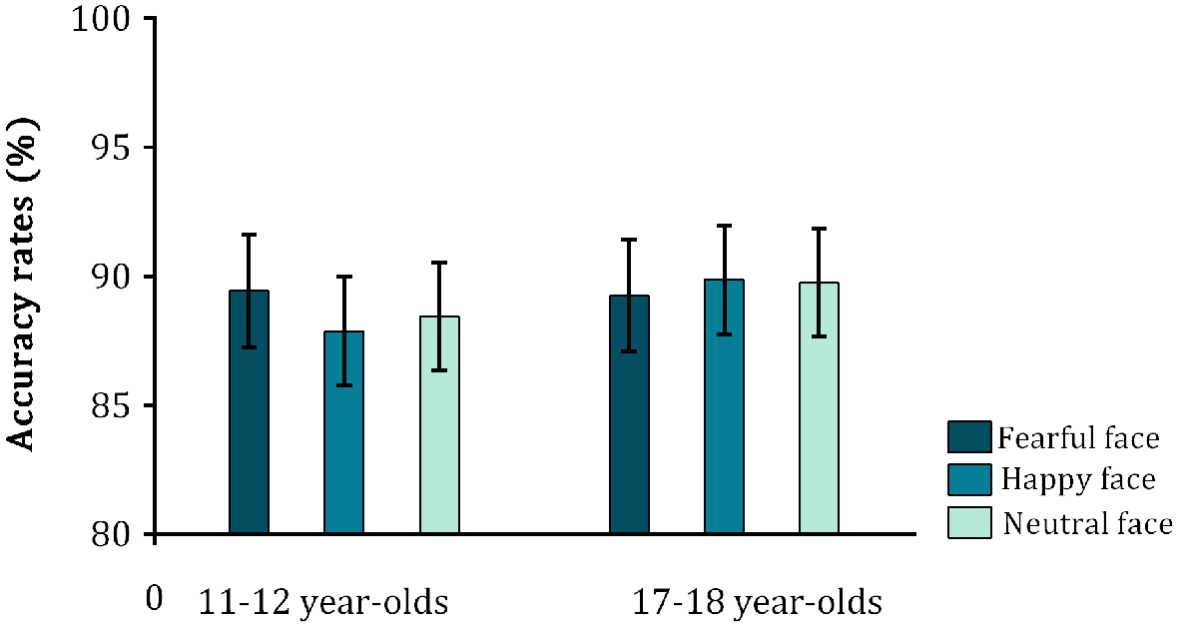
**Mean accuracy rates (in percent) for the go trials in both age groups**. Error bars indicate 1 SE of the mean. None of the main effects, or the interaction was significant.

We then analyzed the accuracy rates for the go and no-go trials, using the same ANCOVA design as for the reaction times above. For the **go trials**, none of the main effects, or the interactions were significant [all *F*s < 1.2, all *p*s = > 0.307] (see Table 1, Figure 3). Moreover, trait anxiety did not correlate with the accuracy rates for go-trials (all *rs* < 0.136, all *ps* > 0.299). Thus, there were no age group or anxiety differences as a function of task performance—and performance across emotional expressions was consistent. We also found that across all participants, RTs and accuracy rates for the go trials were not correlated, thus excluding the possibility of a speed-accuracy trade-off [*r*_s(60)_ = 0.120, *p* = 0.362].

**Figure 4:**
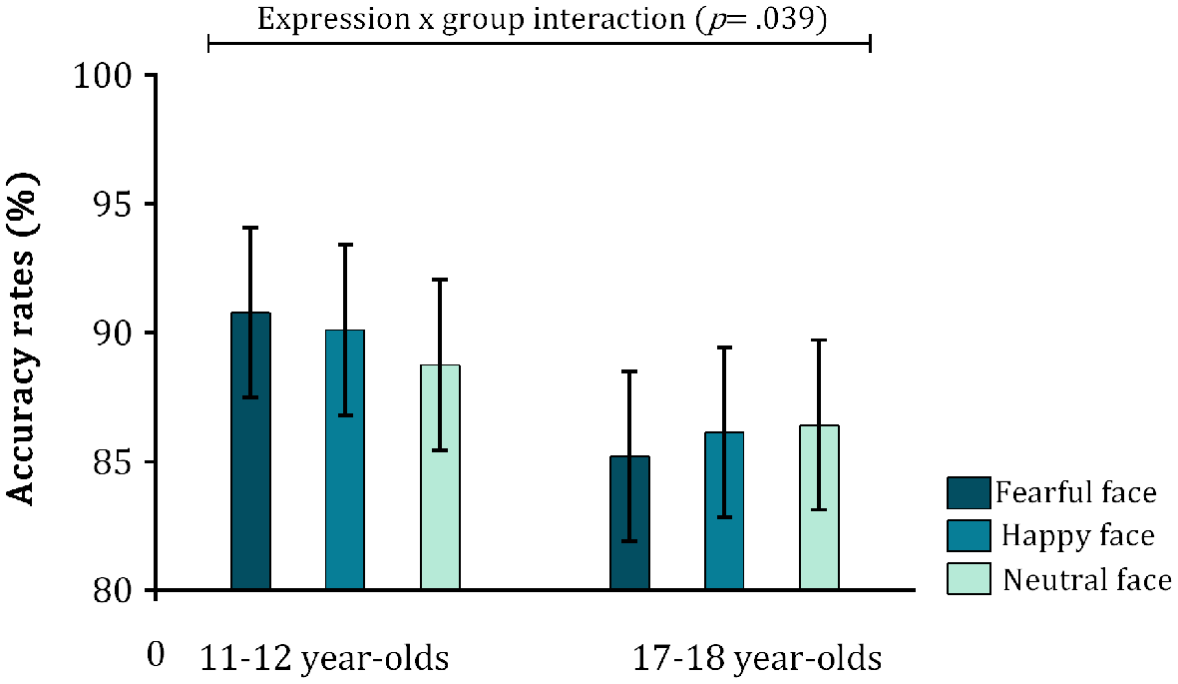
**Mean accuracy rates (in percent) for the no-go trials in both age groups**. Error bars indicate 1 SE of the mean. None of the main effects of expression within age-group or between age-group differences across emotions were significant.

As a secondary aim, we then analysed the effect of age group on expression processing in the no-go trials. Note that while go trial accuracies represent an overt response to a correctly identified target, no-go trial accuracies refer to a *correctly inhibited* overt response to a target (i.e., the absence of a response). For the **no-go trials**, the interaction between expression × age group was significant [*F*_(2, 114)_ = 3.34, *p* = 0.039, η*p*^2^ = 0.055], as was the expression × anxiety interaction [*F*_(2, 114)_ = 3.40, *p* = 0.037, η*p*^2^ = 0.056], and the main effect of expression was significant at trend level [*F*_(2, 114)_ = 2.89, *p* = 0.060, η*p*^2^ = 0.048] (see Table 1 for all effects). However, further decomposition of the interaction between expression and age group for each group separately (Figure 4) established that the simple main effect of emotional expression was not significant in either age group [Younger group: [*F*_(2, 56)_ = 1.06, *p* = 0.057, η*p*^2^ = 0.047]; Older group: [*F*_(2, 56)_ = 1.09, *p* = 0.343, η*p*^2^ = 0.020]. Looking at between-group differences for each emotional expression, we also found no age-group differences for any of the three expressions (all *t*s < 0.393, all *p*s > 0.696). Finally, decomposing the interaction between expression and trait anxiety showed that anxiety scores did not correlate with no-go trial accuracy rates for either emotional expression (all *rs* < 0.085, all *ps* > 0.516).

## Discussion

In the current study we first sought to establish the use of the go/no-go Overlap task as a behavioral measure for assessing differences in attention control to facial expressions at different points across adolescence and across trait anxiety levels. We were able to show that both age groups were able to perform the task at comparable levels. Specifically, there was no difference in the accuracy rates between the two groups for go trials and both groups performed at minimum accuracy levels of 85% or higher. This confirms that the Overlap task can be used successfully in younger adolescents from the age of 11 years, in particular to probe more subtle difficulties in attention control that may be present in reaction times, when faced with the task of deploying attention away from task-irrelevant emotional stimuli. We also found that there were no age-associated differences on no-go trials, which probed the capacity to inhibit motor responses.

Given this, we used the reaction time data on correct go trials to address two specific aims: (i) to assess age-dependent differences in the control of attention toward emotional stimuli (fearful, happy, and neutral faces) across adolescence, and (ii) the extent to which attention control was correlated with trait anxiety in adolescence. We were able to address our question of age-dependent differences in attentional control capacity by comparing a group of younger (11–12 year old) and older (17–18 year old) adolescents in their reaction times during the performance of a primary task while disengaging attention away from distractor stimuli (fearful, happy, and neutral faces). In our study, the younger adolescents exhibited significantly increased response times when disengaging from all expressions during go trials, indicating poorer attention control in the presence of emotional stimuli (that is, faces bearing different emotional expressions). This finding is in line with growing evidence that the successful transition from adolescence to adulthood involves maturing capacity to regulate attention when emotionally evocative, attention-grabbing events occur, particularly in social domains (Monk et al., 2003). However, this poorer attention control was particularly apparent for fearful faces, for which disengagement times were significantly increased in comparison to happy or neutral faces (i.e., main effect of age group on reaction times). This result might be an indication of the increased emotional valence of fearful expressions, and is in line with findings that recognition of emotional expressions emerges over development, with fear recognised much later then happiness (Camras and Allison, 1985). Furthermore, our findings support evidence for the prolonged acquisition of fear-specialised processing abilities during adolescence, (Thomas et al., 2007), including neuroimaging evidence that maturation of emotional processing of fearful, but not happy faces during adolescence is related to progressive acquisition of greater functional activity within the prefrontal cortex (Yurgelun-Todd and Killgore, 2006). We note that these effects generally held when we looked at age-specific differences in attentional control without including anxiety differences as a covariate. Specifically, these additional analyses yielded effects in the same direction as the main analyses, i.e., we found a main effect of age group and a trend toward an interaction between emotional expression and age group.

To test our second hypothesis, that anxiety-proneness would be correlated with attention control capacity in all adolescents, trait anxiety was included as a covariate in all analyses. We found that adolescents (both younger and older) with higher trait anxiety scores were quicker at responding to all probes. This suggests that rather than struggling to usefully deploy attention away from a distracting secondary task, high trait anxiety levels in adolescence can speed up disengagement from face emotion stimuli (independent of slower disengagement times at a younger age)^2^. Our results may reflect an attentional avoidant tendency away from all face emotions in anxious individuals, which at first glance is inconsistent with existing literature that documents attention vigilance toward threatening faces. However, patterns of avoidant responses toward threatening stimuli have been reported in anxious youth under long exposure times of such stimuli (see Lau et al., 2012 for a review). Why these avoidant tendencies emerge to all face emotions rather than just fearful faces in the present study remains unclear. It may be that anxious adolescents are more likely to appraise neutral and happy stimuli in negative ways, leading to greater avoidance of these faces. It is clear that our understanding of the developmental trajectories of anxiety-associated biases and which stimuli they emerge to during adolescence remains sketchy. Nonetheless, recent approaches that investigate the suitability of using attentional bias modification to train anxious children might be the right way forward (Eldar et al., 2011).

Further insights could come from a better understanding of the developmental trajectory of maturing brain networks that support age-related changes in emotion regulation strategies (Blakemore and Choudhury, 2006). More particularly, late development of dorsolateral regions of the prefrontal cortex which have been linked to attention control may explain why this capacity emerges relatively late in adolescence. It has been suggested that investigating developmental changes is akin to aiming at a moving target (Cohen Kadosh, 2011b; Cohen Kadosh et al., 2013b), as not only do cognitive abilities develop, but brain function and structure as well. Future research should therefore adopt multi-level approaches to reveal the interactive relationship between these different factors and to help us understand both quantitative and qualitative changes during development. A better understanding of the factors that support emotion regulation in typically developing adolescents could help us determine sensitive periods during which interventions might be most fruitful (Cohen Kadosh et al., 2013b). Finally, examining whether these abilities are sensitive to variations in anxiety-proneness could help us to target these interventions at particular individuals.

## Conclusions

The current research set out to investigate the role of attentional control capacities in the processing of emotional expressions during adolescence. By using the go/no-go Overlap task for the first time with an adolescent population, we were able to show that younger adolescents, but not older adolescents exhibit more difficulties with attentional disengagement in the presence of emotional faces. Moreover, across groups, adolescents with higher trait anxiety showed an attentional avoidance of all faces, which facilitated relatively better performance on the primary task. A better understanding of how attentional control abilities and emotion processing skills interact during development is clearly important (Crone, 2009), because this will have a knock-on effect on behavior downstream. That is, together they will shape learning about the environment, as well as the acquisition of behavioral response patterns, which could turn out to be maladaptive.

### Conflict of interest statement

The authors declare that the research was conducted in the absence of any commercial or financial relationships that could be construed as a potential conflict of interest.

## Footnotes

^1^Development of the NimStim Face Stimulus Set was overseen by Nim Tottenham and supported by the John D. and Catherine T. MacArthur Foundation Research Network on Early Experience and Brain Development.

^2^We note that gender distribution was unequal in the two age groups, which may have affected these results. Additional analyses however showed that main interaction for RTs remained significant when we included gender as a covariate (Expression × age group [*F*_(2, 112)_ = 3.04, *p* = 0.052, η*p*^2^ = 0.050]; expression × gender [*F*_(2, 112)_ = 0.192, *p* = 0.825, η*p*^2^ < 0.01]). Further, anxiety rates did not differ for the two genders [χ^2^(1, *N* = 60) = 23.17, *p* = 0.726].

## Supplementary material

Table S1 **Analysis of variance (ANOVA) for the factors expression × Age group**.
