# Supplementary material for: Age-related changes in attentional control across adolescence: how does this impact emotion regulation capacities?
Source: Front Psychol. 2014 Feb 12;5:111. doi: 10.3389/fpsyg.2014.00111 (PMC3921606; doi:10.3389/fpsyg.2014.00111)
Supplement: Table S1 — Analysis of variance (ANOVA) for the factors expression × Age group. [file DataSheet1.PDF]

Table S1. Analysis of variance (ANOVA) for the Factors Expression x Age Group

Effect	Reaction times	Accuracy rates go trials	Accuracy rates no-go trials
**Expression**	**F(2,114)= 3.66, p= .029, ηp^2= .059**	F(2,114)= .301, p= .741, ηp^2< .001	F(2,114)= .403, p= .669, ηp^2< .001
**Age group**	**F(1,57)= 8.74, p= .004, ηp^2= .130**	F(1,57)= .378, p= .541, ηp^2< .001	F(1,57)= .374, p= .543, ηp^2< .001
**Expression x age group**	F(2,114)= 2.69, p= .072, ηp^2= .044	F(2,114)= 1.93, p= .150, ηp^2= .032	F(2,114)= 1.44, p= .241, ηp^2= .024
